# Supplementary material for: Isoniazid preventive therapy during infancy does not adversely affect growth among HIV-exposed uninfected children: Secondary analysis of data from a randomized controlled trial
Source: PLoS One. 2024 Aug 16;19(8):e0293708. doi: 10.1371/journal.pone.0293708 (PMC11329125; doi:10.1371/journal.pone.0293708)
Supplement: S1 Table — (DOCX) [file pone.0293708.s001.docx]

## Supplemental table

## S1 Table 1: Factors associated with growth at ~14 and 24-month age

| Follow-up | Variable | **WAZ**^¶^ | | | **HAZ^¥^** | | | **WHZ^ꬰ^** | | |
| --- | --- | --- | --- | --- | --- | --- | --- | --- | --- | --- |
|  |  | **cCoefficient**  **(95% CI)^¤^** | **aCoefficient* (95% CI)** | P-value | **cCoefficient**  **(95% CI)** | **aCoefficient***  **(95% CI)** | P-value | **cCoefficient**  **(95% CI)** | **aCoefficient* (95% CI)** | P-value |
| One point: ~14 months of age | Infant in IPT-arm^ꞩ^ | -0.01 (-0.29, 0.25) | -0.05 (-0.32, 0.21) | 0.686 | 0.13 (-0.17, 0.42) | 0.06 (-0.23, 0.36) | 0.678 | -0.11 (-0.42, 0.20) | -0.12 (-0.44, 0.20) | 0.449 |
|  | Male infants | -0.15 (-0.42, 0.11) | -0.25 (-0.52, 0.02) | 0.071 | -0.52 (-0.81, -0.23) | **-0.53 (-0.83, -0.23)** | **0.001** | 0.11 (-0.21,0.42) | 0.04 (-0.29, 0.36) | 0.830 |
|  | Secondary school or above | 0.21 (-0.06, 0.47) | 0.22 (-0.05, 0.48) | 0.108 | 0.05 (-0.24, 0.35) | 0.01 (-0.28, 0.31) | 0.943 | 0.25 (-0.07, 0.57) | 0.29 (-0.02, 0.61) | 0.071 |
|  | Age of mothers | 0.00 (-0.03, 0.02) | -0.00 (-0.03, 0.02) | 0.794 | -0.02 (-0.05, 0.00) | -0.02 (-0.06, 0.01) | 0.107 | 0.01 (-0.2, 0.04) | 0.01 (-0.02, 0.61) | 0.521 |
|  | Tested HIV positive during pregnancy | 0.03 (-0.28, 0.34) | 0.04 (-0.30, 0.38) | 0.822 | -0.25 (-0.60, 0.10) | -0.31 (-0.69, 0.06) | 0.102 | 0.21 (-0.17, 0.58) | 0.25 (-0.16, 0.66) | 0.233 |
|  | Pregnancy-IPT | 0.27 (0.01, 0.53) | 0.24 (-0.02, 0.51) | 0.075 | -0.01 (-0.30, 0.29) | 0.01 (-0.28, 0.31) | 0.923 | 0.37 (0.06, 0.69) | **0.35 (0.03, 0.67)** | **0.033** |
|  | Suppressed viral load (<40 copies/ml) | 0.08 (-0.27, 0.42) | 0.04 (-0.30, 0.38) | 0.827 | -0.26 (-0.64, 0.12) | -0.19 (-0.57, 0.19) | 0.322 | 0.27 (-0.14, 0.69) | 0.19 (-0.22, 0.60) | 0.359 |
|  | WAZ at birth | 0.24 (0.12, 0.37) | **0.26 (0.13, 0.39)** | **<0.001** |  |  |  | 0.24 (0.09, 0.39) | 0.24 (0.08, 0.39) | 0.003 |
| One point: 24 months of age | Infant in IPT-arm | 0.19 (-0.08, 0.46) | 0.10 (-0.17, 0.37) | 0.468 | -0.00 (-0.35, 0.35) | 0.01 (-0.36, 0.38) | 0.952 | 0.25 (-0.13, 0.63) | 0.12 (-0.25, 0.50) | 0.512 |
|  | Male infants | 0.01 (-0.26, 0.27) | -0.09 (-0.36, 0.18) | 0.493 | -0.02 (-0.37, 0.34) | 0.00 (-0.37, 0.37) | 0.999 | 0.00 (-0.38, 0.37) | -0.11 (-0.49, 0.28) | 0.726 |
|  | Secondary school or above | 0.25 (-0.00, 0.51) | **0.26 (0.00, 0.52)** | **0.050** | 0.31 (-0.06, 0.67) | **0.38 (0.00, 0.75)** | **0.048** | 0.10 (-0.27, 0.48) | 0.07 (-0.31, 0.44) | 0.726 |
|  | Age of mothers | 0.01 (-0.02, 0.04) | 0.02 (-0.01, 0.05) | 0.173 | -0.02 (-0.06, 0.02) | -0.02 (-0.06, 0.02) | 0.334 | 0.03 (-0.01, 0.07) | **0.04 (0.01, 0.08)** | **0.023** |
|  | Tested HIV positive during pregnancy | 0.01 (-0.33, 0.35) | 0.13 (-0.23, 0.50) | 0.473 | -0.14 (-0.58, 0.29) | -0.08 (-0.56, 0.40) | 0.745 | 0.17 (-0.29, 0.63) | 0.28 (-0.21, 0.77) | 0.260 |
|  | Pregnancy-IPT | -0.06 (-0.33, 0.21) | -0.06 (-0.34, 0.21) | 0.639 | -0.13 (-0.48, 0.22) | -0.07 (-0.43, 0.30) | 0.709 | 0.10 (-0.29, 0.49) | 0.05 (-0.33, 0.44) | 0.787 |
|  | Suppressed viral load (<40 copies/ml) | -0.26 (-0.60, 0.07) | -0.31 (-0.65, 0.03) | 0.070 | -0.26 (-0.70, 0.18) | -0.27 (-0.71, 0.17) | 0.231 | -0.17 (-0.63, 0.29) | -0.22 (-0.68, 0.25) | 0.357 |
|  | WAZ at birth | 0.16 (0.03, 0.29) | **0.18 (0.05, 0.31)** | **0.008** |  |  |  | 0.14 (-0.04, 0.32) | 0.15 (-0.03, 0.34) | 0.101 |

^¶^WAZ – weight-for-age. ^¥^HAZ – height-for-age. ^ꬰ^WHZ – weight-for-height z-score. ^ꞩ^IPT – Isoniazid preventive therapy. **^¤^**cCoefficient - crude coefficient. *aCoefficient – adjusted coefficient for all other variables (infant IPT-arm, sex of infants, maternal education, age of mothers in years, timing of maternal HIV diagnosis, maternal IPT use during pregnancy, viral load, WAZ at baseline.
