# Supplementary material for: Isoniazid preventive therapy during infancy does not adversely affect growth among HIV-exposed uninfected children: Secondary analysis of data from a randomized controlled trial
Source: PLoS One. 2024 Aug 16;19(8):e0293708. doi: 10.1371/journal.pone.0293708 (PMC11329125; doi:10.1371/journal.pone.0293708)
Supplement: Attachment — Submitted filename: Response to reviewers_Cherkos.docx [file pone.0293708.s002.docx]

PONE-D-23-31679

Isoniazid preventive therapy during infancy does not adversely effect growth among HIV-exposed uninfected children: secondary analysis of data from a randomized controlled trial

PLOS ONE

Journal Requirements:

When submitting your revision, we need you to address these additional requirements.

1. Please ensure that your manuscript meets PLOS ONE's style requirements, including those for file naming. The PLOS ONE style templates can be found at
   https://journals.plos.org/plosone/s/file?id=wjVg/PLOSOne_formatting_sample_main_body.pdf and <https://journals.plos.org/plosone/s/file?id=ba62/PLOSOne_formatting_sample_title_authors_affiliations.pdf>

**Response: Edited accordingly.**

2. Note from Emily Chenette, Editor in Chief of PLOS ONE, and Iain Hrynaszkiewicz, Director of Open Research Solutions at PLOS: Did you know that depositing data in a repository is associated with up to a 25% citation advantage (https://doi.org/10.1371/journal.pone.0230416)? If you've not already done so, consider depositing your raw data in a repository to ensure your work is read, appreciated and cited by the largest possible audience. You'll also earn an Accessible Data icon on your published paper if you deposit your data in any participating repository (<https://plos.org/open-science/open-data/#accessible-data>).

**Response: Due to the confidentiality agreement the participants signed, the data cannot be deposited, and de-identified data will be provided upon request.**

3. Please note that funding information should not appear in any section or other areas of your manuscript. We will only publish funding information present in the Funding Statement section of the online submission form. Please remove any funding-related text from the manuscript.

**Response: We have removed funding-related text from the manuscript.**

4. We note that the grant information you provided in the 'Funding Information' and 'Financial Disclosure' sections do not match. When you resubmit, please ensure that you provide the correct grant numbers for the awards you received for your study in the 'Funding Information' section.

**Response: We have corrected grant numbers as noted.**

5. Thank you for stating the following financial disclosure: "This work was supported by the Thrasher Research Fund, National Institute of Allergy and Infectious Diseases (NIAID), Fulbright program awarded to the Northern Pacific Global Health Fellows Program by the Fogarty International Center of the National Institutes of Health (NIH/Fogarty), and National Center for Advancing Translational Sciences at National Institutes of Health (NIH) (Thrasher to GJ-S, NIH/NIAID K23AI120793 to SML, NIH/NIAID 2K24AI137310 to TRH, NIH/Fogarty R25TW009345 to AJW and NIH UL1TR000423 for REDCap). ASC had a diversity supplement grant from NIH (NIH/NIAID 1R01AI142647)."  Please state what role the funders took in the study.

**Response: We would like to change the above content by “***This work was supported by the Thrasher Research Fund, National Institute of Allergy and Infectious Diseases (NIAID), and National Center for Advancing Translational Sciences at National Institutes of Health (NIH) (Thrasher to GJ-S, NIH/NIAID K23AI120793 to SML). ASC had a diversity supplement grant from NIH (NIH/NIAID 1R01AI142647). The funders had no role in study design, data collection and analysis, decision to publish, or preparation of the manuscript. The funders had no role in study design, data collection and analysis, decision to publish, or preparation of the manuscript."*

6. We note that you have indicated that there are restrictions to data sharing for this study. PLOS only allows data to be available upon request if there are legal or ethical restrictions on sharing data publicly. For more information on unacceptable data access restrictions, please see http://journals.plos.org/plosone/s/data-availability#loc-unacceptable-data-access-restrictions.

Before we proceed with your manuscript, please address the following prompts:

a) If there are ethical or legal restrictions on sharing a de-identified data set, please explain them in detail (e.g., data contain potentially identifying or sensitive patient information, data are owned by a third-party organization, etc.) and who has imposed them (e.g., a Research Ethics Committee or Institutional Review Board, etc.). Please also provide contact information for a data access committee, ethics committee, or other institutional body to which data requests may be sent.

**Response: We would confirm with the Kenyatta National Hospital-University of Nairobi ERC prior to provision of de-identified data. Interested individuals should contact,**

**STEPHANIE EDLUND-CHO, MSW**
Program Operations Specialist | Global WACh
Department of Global Health 

Hans Rosling Center Box 351620
3980 15^th^ Ave NE, Seattle, WA 98195
office: 206.685.6809
[globalwach.org](https://nam04.safelinks.protection.outlook.com/?url=https%3A%2F%2Furldefense.com%2Fv3%2F__http%3A%2Fwww.globalwach.org%2F__%3B!!K-Hz7m0Vt54!grMcfQkRPSD5kX1ElrvQ9uvJ5Qfc-zcU6S1mFNwNjBKBUionuiIqUYiwvN6LKPfgbIehVKiXMw%24&data=05%7C02%7Cashenafi.cherkos%40unthsc.edu%7C8d3e1bfe428b4d7d186c08dc49d2ab61%7C70de199207c6480fa318a1afcba03983%7C0%7C0%7C638466416404007426%7CUnknown%7CTWFpbGZsb3d8eyJWIjoiMC4wLjAwMDAiLCJQIjoiV2luMzIiLCJBTiI6Ik1haWwiLCJXVCI6Mn0%3D%7C0%7C%7C%7C&sdata=pyFKXKaR8Xj6Cz2vEZk3sgPkEbHpoijv3FET15m25MY%3D&reserved=0)

b) If there are no restrictions, please upload the minimal anonymized data set necessary to replicate your study findings to a stable, public repository and provide us with the relevant URLs, DOIs, or accession numbers. For a list of recommended repositories, please see
   https://journals.plos.org/plosone/s/recommended-repositories. You also have the option of uploading the data as Supporting Information files, but we would recommend depositing data directly to a data repository if possible. We will update your Data Availability statement on your behalf to reflect the information you provide.

**Response: see 6a.**

7. Please include captions for your Supporting Information files at the end of your manuscript, and update any in-text citations to match accordingly. Please see our Supporting Information guidelines for more information: <http://journals.plos.org/plosone/s/supporting-information>.

## Response: added “Supplementary document: S1 Table 1: Factors associated with growth at ~14 and 24-month age*"*

8. Please review your reference list to ensure that it is complete and correct. If you have cited papers that have been retracted, please include the rationale for doing so in the manuscript text, or remove these references and replace them with relevant current references. Any changes to the reference list should be mentioned in the rebuttal letter that accompanies your revised manuscript. If you need to cite a retracted article, indicate the article's retracted status in the References list and also include a citation and full reference for the retraction notice.

[Note: HTML markup is below. Please do not edit.]

Reviewers' comments:

Reviewer's Responses to Questions

**Comments to the Author**

1. Is the manuscript technically sound, and do the data support the conclusions?

The manuscript must describe a technically sound piece of scientific research with data that supports the conclusions. Experiments must have been conducted rigorously, with appropriate controls, replication, and sample sizes. The conclusions must be drawn appropriately based on the data presented.

Reviewer #1: Yes

Reviewer #2: Yes

2. Has the statistical analysis been performed appropriately and rigorously?

Reviewer #1: I Don't Know

Reviewer #2: Yes

3. Have the authors made all data underlying the findings in their manuscript fully available?

The [PLOS Data policy](http://www.plosone.org/static/policies.action#sharing) requires authors to make all data underlying the findings described in their manuscript fully available without restriction, with rare exception (please refer to the Data Availability Statement in the manuscript PDF file). The data should be provided as part of the manuscript or its supporting information, or deposited to a public repository. For example, in addition to summary statistics, the data points behind means, medians and variance measures should be available. If there are restrictions on publicly sharing data—e.g. participant privacy or use of data from a third party—those must be specified.

Reviewer #1: Yes

Reviewer #2: No

4. Is the manuscript presented in an intelligible fashion and written in standard English?

PLOS ONE does not copyedit accepted manuscripts, so the language in submitted articles must be clear, correct, and unambiguous. Any typographical or grammatical errors should be corrected at revision, so please note any specific errors here.

Reviewer #1: Yes

Reviewer #2: Yes

5. Review Comments to the Author

Please use the space provided to explain your answers to the questions above. You may also include additional comments for the author, including concerns about dual publication, research ethics, or publication ethics. (Please upload your review as an attachment if it exceeds 20,000 characters)

Reviewer #1: General
This is a clear well written and informative paper. It is easy to follow and understand and the authors clearly demonstrate why the study was carried out, its importance, how the study was carried out, the findings/results and how the results were interpreted. The study question is answered appropriately.
In this study the authors do a secondary data analysis on data collected during a randomized control trial and compare the growth outcomes of HIV exposed uninfected (HEU) infants who received isoniazid preventive therapy (IPT) for the first year of their life with those who did not receive any IPT. They found that IPT did nit significantly affect the growth outcomes of infants in the first two years of their life.

Abstract
The authors summarise the main research question and key findings well

Introduction
The authors give us the importance of the study - and they explain about the added advantage of leveraging on the RTC design to be able to investigate the effect of IPT on growth of HEU infants without introducing the confounding brought about by other factors such as known exposure to TB.

Figures and tables
The bottom right Lowess curve is mislabelled. it is labelled "predicted height-for-age, adjusted for baseline HAZ" instead of "predicted weight-for-height, adjusted for baseline WHZ".

**Response: Thanks for noticing this error. We have corrected the mislabeled noted.**

Methods
The parent trial and design and intervention is explained well. Ethical protocols were observed.
However, the specific tools that were used to measure the infant's weight and height are not described.
Statistical methods – The authors could perhaps mention why they use LMEMs with autoregression correlation structure and what it entails.
The authors explain how they dealt with missingness which is good.

**Response: We used conventional growth measurement tools – a scale for weight and measuring tape for height.** **All data collectors were trained by the CDC-Kenya team on how to measure an infant's weight and height.**

Results, discussion, conclusion
Generally, the results support the conclusion. Limitations are discussed appropriately. Previous studies and supporting data is shared.
However, the information in line 245 – 248 somewhat seems misplaced and confusing. It does not quite make sense how the mortality of male infants is explained by the value placed on females in the society and how this is all related to the observed finding of declining HAZ in male infants.

**Response: edited and added “**The biological and social mechanisms driving higher neonatal mortality and morbidity in males and their greater HAZ decline compared to females remain undefined (34,35), leaving the cause of this disparity unclear.”

Reviewer #2: Isoniazid preventive therapy during infancy does not adversely effect growth among HIV-exposed uninfected children: secondary analysis of data from a randomized controlled trial

Summary
This is a secondary analysis of data from a randomized controlled trial assessing growth among HIV exposed uninfected children in Western Kenya with and without INH prophylaxis. There were no differences in growth between children receiving INH and those who did not. Significant stunting was observed in both arms by 24 months of age.

Comments
General: This is an important study showing the safety of INH prophylaxis initiated in infants from 6-10 weeks of age. While the benefits of this prophylaxis are still under study as demonstrated in the primary study and its limitations, the current study resolves the question of safety with specific focus on growth indicators.

Specific comments:
Title - Consider replacing "effect" with "affect" as "adversely effect" seems to imply causing something that was not expected or desired whereas "adversely affect" implies altering an expected process to a less desirable or undesirable state.

**Response: Replaced "effect" with "affect."**

Line 3: Consider rephrasing to "Without treatment, children with latent Mtb infection (LTBI) have about…" as it is not true that Mtb infection is also called latent TB infection.

**Response: Thanks for the comment: We edited it accordingly: "Without treatment, children with latent Mtb infection, also called latent TB infection (LTBI)"**

Line 19-20: there is a missing closing parenthesis.

**Response: Thanks for noting this. The closing parenthesis is added.**

Infant growth characterization: Consider adding more detail on the tools and methods used for measurement of height and weight as these are the main measurements for this study. Did all the clinics use similar tools?
Are these clinics in urban or rural areas and are they public health clinics?

**Response: All data collectors were trained by the CDC-Kenya team on how to measure an infant's weight and height. Under the participants and study period subheading, we added: "All institutions in the study were public maternal and child health (MCH) clinics embedded in Kenya's Ministry of Health (MOH)."**

Line 121: Rephrase to "All participating mothers were living with HIV…"

**Response: Thanks for the comment: we changed it accordingly.**

Table 2: Consider standardizing placement of symbols for WAZ, HAZ, and WHZ

**Response: Thank you for the comment: placement standardized.**

Line 228: There's a missing period.

**Response: Thank you for the comment. Period added.**

Lines 237 – 242: Is this proportion of stunting consistent with other studies of HEU children? Consider citations https://www.sciencedirect.com/science/article/pii/S2405844019357330, https://www.ncbi.nlm.nih.gov/pmc/articles/PMC7868486/, <https://onlinelibrary.wiley.com/doi/full/10.1002/fsn3.2509>

**Response: We cited two of the recommended papers that have follow-up periods similar to ours. We added, "Similarly, studies conducted in Kenya (28) and Zimbabwe (29) found that 38.9% and 30% of HEU children are severely stunted, respectively."**

6. PLOS authors have the option to publish the peer review history of their article ([what does this mean?](https://journals.plos.org/plosone/s/editorial-and-peer-review-process#loc-peer-review-history)). If published, this will include your full peer review and any attached files.

If you choose "no", your identity will remain anonymous but your review may still be made public.

**Do you want your identity to be public for this peer review?** For information about this choice, including consent withdrawal, please see our [Privacy Policy](https://www.plos.org/privacy-policy).

Reviewer #1: **Yes:** Linda Mandikiyana Chirimuta

Reviewer #2: No
